# “What kind of general practitioner do I need for smoking cessation?” Results from a qualitative study in Poland

**DOI:** 10.1186/1471-2296-14-159

**Published:** 2013-10-20

**Authors:** Krzysztof Buczkowski, Ludmila Marcinowicz, Slawomir Czachowski, Elwira Piszczek, Agnieszka Sowinska

**Affiliations:** 1 Department of Family Medicine, Collegium Medicum, Nicolaus Copernicus University, Sklodowskiej-Curie 9, 85-094, Bydgoszcz Torun, Poland; 2 Department of Family Medicine and Community Nursing, Medical University of Bialystok, Mieszka I 4B, 15-054, Bialystok, Poland; 3 Sociology Institute, Nicolaus Copernicus University, ul. Fosa Staromiejska 1a, 87-100, Torun, Poland; 4 Department of English, Nicolaus Copernicus University, ul. W. Bojarskiego 1, 87-100, Torun, Poland

**Keywords:** Smoking cessation, Primary care, Qualitative research

## Abstract

**Background:**

Cigarette smoking remains the leading preventable cause of death and disease. Thus, all activities aiming to reduce smoking play an important role in improving population health. The positive role of the general practitioner (GP) in smoking cessation could increase the success rate for quitting smoking, if compared with unassisted cessation. The aim of this study was to determine what kind of general practitioner smokers need in order to stop smoking.

**Methods:**

Four focus groups with 12 current and 12 former smokers (aged 20-59, 11 women and 13 men), were arranged in the city of Toruń, Poland, with a view to describe their opinions on the GP’s role in smoking cessation. The data were subjected to descriptive qualitative content analysis.

**Results:**

Two major themes emerged in the analysis: the smokers’ positive and negative experiences of the GP in smoking cessation and their expectations regarding the role of the GP in smoking cessation. The first theme embraced the following subthemes: (1) GP’s passivity, (2) routine questions about the patient’s smoking during the visit, (3) lack of time during the visit, and (4) the role model of the GP in smoking cessation. Within the second theme, the respondents identified the following subthemes: (1) bringing up the topic of smoking cessation, even in situations when the patient is unprepared for this; (2) the necessity of a tailored approach to the patient; (3) access to information and evidence confirming the harms of smoking tobacco; (4) prescription of pharmacological and other treatment; and (5) referral to specialists in smoking cessation.

**Conclusions:**

Patients expect their GP to actively participate in smoking cessation through a more tailored approach to the patient’s needs. The patients’ experiences did not match their expectations: the smokers rarely got advice on smoking cessation from their GPs. Finally, they emphasized the importance of the GP as a role model in smoking cessation.

## Background

Cigarette smoking remains the leading preventable cause of death and disease across the world [1,2]. The World Health Organization (WHO) estimates that globally over one billion people smoke tobacco [3]. Furthermore, it is estimated that 21% of cancers are due to smoking [4] and that by 2015 smoking will be the cause of 10% of all deaths [5]. The WHO also attributes approximately six million deaths per year to tobacco, and this is expected to rise to around ten million per year by 2030 [6].

Smokers now represent close to 29% of the adult population in Poland [7]. But in the past consumption of cigarettes, especially in the 1970s and 1980s, was one of the highest in the world, reaching approximately 3600 cigarettes per adult [8]. Since the mid-1990s, the sale and consumption of cigarettes have declined.

Cross-sectional studies show that most smokers in developed countries want to stop and intend to stop at some point [9]. The increasing number of people who want to stop smoking plays a key role in improving population health, and, for this reason, helping smokers to quit is one of the most cost-effective medical interventions available [10,11].

Reducing the prevalence of smoking remains one of the most important public health goals [12,13]. To this purpose, in 2010 Polish parliament approved Act of Amendment of the Act on Protection of Health Against the Consequences of Consumption of Tobacco and Tobacco Products [14]. It introduced a ban on smoking in public places, a ban on tobacco advertisements and the requirement of health warning labels on tobacco products [15]. Furthermore, the government takes actions in order to increase the price of cigarettes, which for the most popular price category (20 cigarettes) is currently 1,92 Euro (7,95 Polish zloty). Excise taxes total 66% of the weigthed average price [16].

Besides, many nationwide campaigns are organized to reduce smoking, for example, World No Tobacco Day, celebrated annually, or the international day of quitting smoking. The Civic Coalition “Tobacco or Health”, founded in 2003, associates a number of non-governmental organizations acting locally to encourage people to stop smoking. An example of a local initiative addressed to current smokers is the campaign “Know the age of your lungs” held in Torun and Bydgoszcz, which offers spirometric examination with anti-nicotine intervention.

In Poland smoking cessation interventions are administered mainly by the GP, and in the case of patients referred for consultation – also by specialists. No gratuitous stop smoking services are available in Poland. If patients want to get help in smoking cessation in primary care, they visit their GP. However, the GP spends most of his time treating patients, not promoting health. Family medicine was implemented in Polish healthcare system only 20 years ago, so its position up to this day isn’t stable. An average GP has approximately 2500 patients at all ages on his list, consults around 40 patients a day, and has 4 home visits [17,18]. Patients have access to their GPs from Monday to Friday from 8.00 am to 6.00 pm. Although many GPs feel overburdened with workload and administrative duties [19], over two-thirds of patients feel satisfied with their GP [20].

Over the last couple of years knowledge about smoking cessation has broadened considerably. Pharmacologic treatments have been developed and access to behavioral treatment improved. In addition, in many countries there are special services which help in the process of stopping [21]. Owing to increased knowledge of smoking harm and public initiatives to limit smoking more and more smokers make attempts to quit, yet in the majority of cases these are spontaneous attempts. The most common method of stopping smoking is still unassisted cessation: cold turkey or reducing before quitting [22,23]. These unassisted activities are effective, however, the chances of successful quitting would increase if attempts were accompanied by pharmacotherapy or behavioral treatment, [24]. Unassisted quit rate is 2-3%. A brief advice intervention can increase quitting by a further 1 to 3%. Adding nicotine replacement therapy (NRT) can increase the rate of quitting by another 0,7-2,1% [25]. Buproprion is of similar efficacy to nicotine replacement [26]. Varenicline increases the chances of successful long-term smoking cessation by two- and threefold [27] and seems to be the most effective pharmacotherapy for smoking cessation [28]. These various forms of pharmacotherapy are available in Poland, including NRT without prescription, but they are non-refundable.

Hence, it is essential that those who are intent on quitting smoking have access to pharmacotherapy and any form of medical help. This can be provided by GPs, especially through motivating smokers and offering smoking cessation support to any smoking patient in relevant clinical situations. The special doctor-patient relationship and frequent visits may help to identify those who ought to quit, but who do not manage on their own.

There are many international [29,30] and national guidelines (designed, for example, by College of Family Physicians in Poland [31]), which help GPs deal with smoking patients. These guidelines recommend combining medication and behavioral support to help people stop smoking. The most frequently recommended intervention is the so-called “five As”: ask, advise, assess, assist, and arrange. This strategy assumes that each time a smoking patient visits the doctor the topic of smoking is mentioned, and that the patients ready to quit are provided with help [30-32]. However, the data show that this procedure is not widely put into practice [33,34]. This is the result of a lack of time for such intervention during routine visits, and the fact that a structured intervention is not entirely accepted by patients, who may expect from their GPs a more tailored approach [23].

The aim of this study was to explore current and former smokers’ experiences and expectations of GPs’ help in smoking cessation in a Polish medical setting.

## Methods

Descriptive qualitative analysis was used with focus group data in order to explore the smokers’ views of their GP’s role in smoking cessation. The method refers to Sandelowski [35] and Lundman&Granheim [36]. This method is orientated towards establishing a straight descriptive summary of the informational contents of the data.

### Design

Since our study deals with sensitive issues that require deep discussion during the interview, which included the respondents’ health, addiction, and doctor-patient relationships, the qualitative method for generating data seemed the most appropriate one to explore the topics. Focus groups were used to collect data as they encourage interaction amongst participants, and highlight areas of agreement or disagreement within a group. Furthermore, the dynamics of focus group discussions could reveal aspects of the smoking cessation issue that were not taken into account when we devised the topic guide (Additional file 1). Finally, focus groups allowed us to investigate the participants’ expectations and beliefs about the role of the GP in smoking cessation, in addition to their experiences of quitting.

### Participants, setting and data collection

Purposive samples of patients for focus groups were recruited through advertisements disseminated at the Institute of Sociology of Nicolaus Copernicus University in Torun (EP) and two general practices in Bydgoszcz and in Torun, Poland (KB and SC). The advertisement at the Institute of Sociology was addressed to everyone willing to participate in the study, irrespective of their age or student status. Advertisements for the research project were also placed in the practices, which together provide medical services to around 15000 patients. In addition, patients were informed about the opportunity to participate in the project by their GP or nurse. The study was conducted between December 2009 and March 2010.

Four focus groups with a sample of 24 patients (12 current and 12 former smokers) were arranged: two groups of current smokers and two of former smokers.

During the recruitment process, the smoking status was defined according to the WHO criteria [37,38]. A patient who had been smoking for at least six months and was a smoker at the time of the study was defined as a current smoker. A person who had smoked for at least six months, and who hadn’t smoked for at least three months prior to when the study was conducted, was regarded as a former smoker. The smokers also had to be 18 years old or older.

After the third focus group, data saturation was achieved. Data saturation refers here to the point at which additional interviews do not provide any new insights into the topic. As one more focus group was already arranged, we elected to continue data collection with this last group.

Eleven women and thirteen men, aged 20–59, participated in the study. The mean age of the participants was 38 years old. The average duration of smoking was about 16 years (range 1–35 years). It was slightly shorter in women (13 years) than in men (almost 17 years). Smoking status and participants’ characteristics are presented in Table 1. While presenting the results, we use the numbers of participants and so, for example, P2 stands for Participant number 2.

**Table 1:** Focus group characteristics

**Item number**	**Focus group number**	**Age**	**Sex**	**Smoking status**	**Duration of smoking (years)**
1	1	20	F	C	3
2	1	29	F	C	10
3	1	35	F	C	20
4	1	53	M	C	25–30
5	1	21	M	C	6
6	1	43	F	C	27
7	2	24	M	C	1
8	2	43	F	C	-
9	2	52	M	C	35
10	2	21	F	C	4
11	2	26	M	C	11
12	2	33	F	C	17
13	3	43	M	F	11
14	3	31	M	F	13
15	3	28	F	F	8
16	3	57	M	F	15
17	3	30	F	F	13
18	3	57	F	F	15
19	3	42	M	F	15
20	4	48	M	F	30
21	4	40	F	F	13
22	4	28	M	F	12
23	4	49	M	F	30
24	4	59	M	F	20

C – current smoker.

F – former smoker.

Discussions were moderated by an experienced facilitator (EP) according to the topic guide derived from the literature, which was adjusted after pilot discussions with two current and former smokers who were excluded from the study. The focus group discussions lasted from 60 to 90 minutes*.* All discussions were tape-recorded and transcribed verbatim.

### Data analysis

The focus group interviews, in the form of recordings and transcriptions, were analysed by a multidisciplinary team (researcher, physician, sociologist, linguist).

Three authors (KB, LM and AS) read the transcripts of focus group interviews to obtain a sense of the whole and become familiar with each focus group interview. Meaning units which described the smokers’ experiences and perception of their GPs’ actions in smoking cessation were next identified, condensed and independently labeled with various codes to express a general understanding of them (Table 2). During the analysis the authors stayed close to the data and the surface of words and events, paying attention to recurring regularities and the words that appeared to capture key thoughts The various codes were subsequently compared and formulated into subthemes and themes, after discussion with other researchers (EP and SC). Two major themes emerged during the analysis, which are described in the following section. Selected quotations, which reflect the smokers’ most typical answers, were then translated into English by the linguist (AS).

**Table 2:** Example of meaning unit, condensed meaning unit, interpretation, subthemes and themes from content analysis

**Meaning Unit**	**Condensed meaning unit, description close to the text**	**Interpretation (Code)**	**Subtheme**	**Theme**
*“[The GP] should treat everyone individually, be able to relate to the patient’s disorders because we wouldn’t care about something that is routine.*	Should treat everyone individually, relate to the patient’s disorders, patients don’t care for routine	The necessity of an individual approach to the patient, lack of routine on the GP’s part is conducive to patient compliance	GP’s tailored approach to the patient	Expectations of the GP’s behaviour

### Ethical considerations

Ethical approval was granted by the Bioethical Committee of the Collegium Medicum at Nicolaus Copernicus University, Bydgoszcz, Poland. Potential participants were provided with information about the study, and those who decided to participate gave an informed consent. Confidentiality was assured and anonymity of the smokers was protected.

## Results

The analysis of the transcripts revealed that the patients’ experiences of their GPs’ behavior were predominantly negative, and resulted either from the GP’s bad practices or the shortcomings in the Polish primary care system. They were divided into the following subthemes: (1) GP’s passivity, (2) routine questions about the patient’s smoking during the visit, (3) lack of time during the visit and, finally, (4) the role model of the GP in smoking cessation.

The experiences show that anti-smoking intervention was rarely undertaken by the GP, and if it was, the GP was not much engaged. The participants appreciated a tailor-made approach, which was evident in their positive response to the GP’s own experience in smoking cessation.

### Patients’ experiences of their GP’s actions in quitting smoking

#### GP’s passivity

Although the patients differed in their experiences, many of them admitted that their GP had never raised the issue of smoking. This stands in stark contrast to the patients’ expectations. The experience of the GP’s passivity was commonplace and is illustrated by the former smokers who succeeded in quitting smoking, even though their GP did not motivate them to do it, neither did he participate in the other stages of smoking cessation.

“So far I haven’t met a doctor who would ever mention smoking, never…” (P13)

“As a patient, I’ve never met with this… that the doctor asked about smoking…” (P21)

These examples indicate that Polish GPs often ignore the problem of smoking during the consultation. Patients do not evaluate this behavior positively. They think that prevention should be part and parcel of the consultation.

“I agree that the GP should help in smoking cessation, explain and answer questions, but should do it out of his own will, and not because I visit him especially and only for this (P17).”

In sum, for various reasons lack of questions about smoking during the consultation was not met with patients’ content.

#### Routine questions

In the few cases where the GP did intervene, the patients drew attention to the doctor’s routine questions, which lacked a personal touch. This behavior had no motivating effect on the smokers and did not encourage them to seek help with their GP. The following examples corroborate this:

“The GP asked me about smoking during the periodic check-up. When I admitted to it, he asked how many, 20, give it up, give it up, and nothing more.” (P 19)

According to the respondents, the GP’s routine behavior caused disappointment and from the perspective of anti-nicotine interventions it was a missed opportunity for building motivation. Likewise, the next example shows that routine questions without any particular information tailored to the patient’s needs and expectations are not welcome by patients, even though the problem of smoking was brought up by the GP during the consultation.

“When I go to the doctor, he also asks me if I smoke or if I’m planning to quit. So routinely… and he says that I should stop smoking, but nothing specific has he told me so far, only to stop smoking… that smoking is harmful, and all that jazz… generally…” (P4)

#### Lack of time during the visit

The respondents pointed to haste and a lack of time to bring up the problem of smoking during the visit as some of the possible causes of lack of GP intervention. Many of the investigated patients noticed that the GP barely had time to resolve basic medical problems. Only a few patients admitted to talking with their GP about quitting smoking, even though the promotion of a healthy lifestyle, if necessary through anti-nicotine intervention, should be a responsibility that lies with the GP.

“Doctors don’t start the topic of smoking… they are constantly in a hurry… They’ve got only a moment for the patient, done, and next please, next…” (P8)

“Frankly speaking, the GP doesn’t necessarily have time for this [to make people aware of the harms of smoking] because we know what’s going on, for example, in the [flu] season… I often go because I’ve got kids, they are ill, and I know what queues there are, how long one sometimes has to wait…” (P3)

According to the respondents, it was precisely lack of time that was a major cause of missing anti-nicotine interventions or other actions during the consultation which did not meet their expectations.

#### The role model of the GP in smoking cessation

What the respondents found relevant was whether their GP used to be a smoker, if he was able himself to comply with the advice he gives to his patients and, finally, what experience he had in smoking cessation activities. One of the most demotivating GP activities found by some patients was their GP’s own smoking habits. Such behavior meant that the doctor was not reliable when encouraging the patient to quit. The belief held by the patients was that, if the GP was not able to help himself, he certainly wouldn’t be able to help them.

“In my health centre, there is no GP who wouldn’t smoke (…) And so if the GP himself smokes he will not advise [me] to quit for health reasons” (P4)

Others mentioned the GP’s inconsistency in giving advice about smoking cessation and an indirect acceptance of smoking, which was demotivating, according to the respondents.

“When I was sitting in the surgery and the GP kept on explaining that I must do this and that, and a moment later he was saying – ‘let’s go, have a smoke’, the motivation disappeared straightway” (P23)

Another negative experience was the smokers’ feeling that the GP does not believe in their success of quitting. This behavior was also regarded as a disincentive for smoking cessation.

“She’s examined me and she’s saying: ‘You won’t quit anyway’, but earlier she told me not to smoke, and then she’s saying ‘You won’t give up anyway, if you’ve been smoking for so many years’ … She didn’t even discourage me.” (P9)

However, there were also a few positive experiences that emerged in the discussions. Showing that smoking cessation may also be the GP’s problem, that the GP too had to make an effort to quit, allows the patient to look on his doctor from a different perspective and enhances the doctor-patient relationship. In the following excerpt the patient points to a convincing example of the GP’s own experience:

“And he [the GP] told me: ‘I used to smoke but freed myself from it, and just gave up cigarettes…’; I found it more convincing that he’d said to me: ‘and I freed myself from it’, not from the medical point of view, but as a human, that he went through it himself; he impressed me.” (P3)

### Smokers’ expectations about the role of the GP in smoking cessation

The smokers expressed expectations of a GP’s active role in smoking cessation, which would involve bringing up the topic of smoking cessation during the consultation, adopting a tailored approach to the patient, showing reliable evidence on the harms of tobacco smoking and prescribing pharmacological or other treatment. Finally, the respondents also emphasized the importance of other specialist services and psychological support.

#### Bringing up the topic of smoking cessation in situations where patients are not prepared to bring it up themselves

Most respondents clearly stressed that the GP should initiate the topic of smoking cessation during the consultation and inform them about health hazards and the possibility of help, rather than wait until patients themselves ask for help. Yet, this is not to happen at every visit, especially when the reason of the consultation is not smoking related.

Patients are often ashamed to admit to smoking and find it difficult to ask the GP for help in quitting. They assume the GP will start the topic of smoking cessation himself, offering the patient adequate help, particularly when they do not feel ready themselves to bring the issue up.

“The best in my case would be if the GP asked me if I want to give up smoking, if I smoke…, then I could possibly ask: What is good and effective advice to quit, Doctor? (P 6)

Some respondents were very pronounced about it and pointed out it is not only the GP’s responsibility to touch upon the issue of stopping smoking, but also manage the patient in such a way as to resolve the problem.

“I think the GP is there to ask and to help. And to see the whole patient and not just because you’ve got a runny nose or you’re cold… the doctor should say ‘I will manage you, come to me every week or once a month.” (P8)

#### The necessity of a tailored approach to the patient

Frequently, the patients reported the need for a tailored approach. In contrast to a routine approach, a more individualized approach that meets the patient’s needs is conducive to the patient’s compliance and thus, in turn, to his attempt to change a lifestyle and give up smoking. However, according to the patients, this approach should also make provision for the cases when the patient is not intent on quitting smoking, and then his decision must be respected and the GP should abstain from any intervention. Still, when the patient struggles on whether to quit or not, the GP’s role in building or reinforcing motivation is crucial.

“[The GP] should treat everyone individually, be able to relate to the patient’s disorders because we wouldn’t care about something that is routine. If we keep on always hearing the same things at the doctor’s, we’ll stop listening. It depends on the way the doctor approaches the patient, his ability to influence the patient…” (P19)

What lies at the heart of this approach is the GP’s willingness to know the patient better and improve the relationship with him, so that he can feel that what the GP prescribes is only for him and results from the GP’s care for his health. One of the patients, who was poorly motivated to quit, emphasized that a tailored approach could have a motivating impact on her:

“If only there was such a tailored approach… If only I felt that this is only for me…maybe I would consider it [giving up smoking]” (P12)

Although the respondents would like their GP to bring forth the topic of smoking cessation during the consultation, even in the case when they are unprepared for this, they also seem to have very specific expectations regarding the GP’s behavior. They do not want their GP to be too aggressive. Such behavior can only deteriorate the relationship between the GP and the patient, and does not have a motivating influence on the patient’s decision about smoking cessation. One of the smokers put it in the following way:

*“I wouldn’t like it [the GP’s approach] to be too insistent… that GPs attack smokers… I’d like to know that in the instance where I do want to quit, the doctor will provide me with help, explain, answer the questions and understand my situation… [The GP] shouldn’t be too aggressive in raising smokers’ awareness… [he] should outline that this and that can cause this and that…”* (P17)

#### Access to information and reliable evidence on the harms of tobacco smoking

When the issue of smoking is brought up during the interview, the investigated patients would expect from their GP reliable evidence confirming the harms of tobacco smoking, and some information detailing effective methods of treatment.

Despite common knowledge that smoking is harmful, it is the GP that was widely expected to provide reliable information. The following examples highlight the role of the GP, which involves providing access to evidence and adequate information about the harms of tobacco smoking:

“The doctor would have to present facts, either using photos or tell stories from his professional career that he used to have such patients and they gave up… or that those who didn’t, for example, are already dead.” (P7)

“If someone professionally explained to me why I smoke, what makes me like smoking…” (P12)

#### Prescription of pharmacotherapy and other treatment

Likewise, in the case of therapy, the GP should be well informed of widely accessible methods of treatment and the means of their application. The investigated patients expressed that they would be happy to obtain information about successful treatment from their GP, especially about effective medicines.

“My GP prescribed me pills for smoking cessation when I asked her… I asked her because my friend gave up smoking in this way. He got a prescription from his GP… and he hasn’t been smoking for 2 months already.” (P9)

Receiving effective treatment was very much expected by the respondents. In particular, they would like to be prescribed pills which would make them stop smoking. This is illustrated by the following example:

“If I knew that there would be some treatment, that is, that I would get medicines, I would be pleased to benefit from that.” (P6)

But some smokers were open to any form of therapy, not only effective pills.

“If my GP suggested some sort of therapy or something like this, I think it would be great…” (P9)

#### Referral to specialists in smoking cessation

Some of the patients expected to be referred to specialist stop-smoking services. This can be explained by greater trust in treatments offered by a specialist in smoking cessation, rather than by the GP. Another reason for this expectation of referral was the opportunity to benefit from psychological help. In the discussions, patients underlined an important role for psychological support in smoking cessation. This is illustrated by the following examples:

“If there is a referral to a particular specialist and there is an illness related to it [smoking], that smoking has impact on… the specialist can somehow show it… that because of that [smoking] it is so, and not otherwise.” (P16)

“The psychologist could intervene… in order to make us start to think differently about smoking and one’s life.” (P18)

## Discussion

This study presents smokers’ experiences and expectations related to the role of the GP in smoking cessation. The main finding of our research is that the patients expected their GP to intervene and bring up the topic of smoking cessation. More specifically, in line with these expectations the GP should adopt a tailored approach to the patient and continue pursuing anti-nicotine interventions; or, if it is the patient’s will, the GP should not mention the issue of smoking at all.

The respondents expected a more person-oriented approach rather than only a routine interview or routine consultation. This is in agreement with other studies, which indicated preferences for a more client-centered approach in general practice [39]. In particular, irrespective of the purpose of the visit, patients’ preferences show that they expect a GP who will have time for them, listen to what they say and advise them on how to get better or how to protect their health in the future [39,40]. This study illustrates that the patients’ expectations also concern the process of smoking cessation. This process must be more tailored, personalized, going beyond standard procedures in order to bring positive effect. A tailored approach facilitates doctor-patient communication: GPs can learn about patients’ reasons for smoking and adjust arguments to build their motivation. When the smoking patient decides to quit, in the tailored approach, both the patient and the GP choose the way of smoking cessation and a form of pharmacotherapy. In short, GPs need to establish a more satisfying therapeutic relationship, individualize treatments to suit the whole person and give up a routine consultation.

The patients also expected to be provided with reliable information. It seems that good information plays a crucial role in motivating them to take actions which lead to a change in lifestyle. An important element in managing smoking cessation, which is also expected by patients, is effective pharmacotherapy. The validity of these expectations is corroborated by numerous studies, which indicate that introducing pharmacotherapy increases the number of patients permanently giving up smoking [25-28,41].

This study has also shown how significant the GP’s example is to those who contemplate quitting. Most participants admitted they would feel more motivated to stop smoking if their GP was a non-smoker. An interesting finding was the patients’ experience of their GP as a role model in smoking cessation. Notably, it was found as particularly persuasive when the GP – the former smoker – presented the problem of quitting tobacco smoking not from a medical perspective, but from the perspective of the smoker. GP’s sharing of his own experience seemed to have a modeling effect on the listening patient. To put it differently, the patient may have possibly been more prone to emulate the GP’s behavior and change his own lifestyle.

Furthermore, some of the patients would expect their GP to firstly motivate them to quit smoking, and then refer them to a specialist in smoking cessation. These expectations are consistent with the results obtained in a study by Wilson et al., in which patients often expected their GP to start the treatment followed by a referral to stop-smoking services [42]. In a different study, Murray et al. showed that the identification of smokers in general practice and subsequent referral to cessation services increased the number of quit attempts [43]. Thus, these actions can produce positive effects in the smoking cessation process. Although the investigated smokers indicated the need for behavioral support, there aren’t any widely available services for smoking cessation in Poland.

In general, the patients’ experiences did not match their expectations: the smokers rarely spoke with their GP about quitting smoking and seldom got advice on smoking cessation. This could be explained by a lack of GP intervention for smoking cessation, but there may also be little or no interest in seeking out help from the patients themselves. The first problem has already appeared in a study of Australian GPs, only a third of whom reported providing cessation advice during every routine consultation with a smoker [33]. The second is partly corroborated by observations on Danish patients and GPs presented in Guassora et al. [44]. Their study revealed that smoking cessation advice shouldn’t be given during every routine consultation with a smoker especially when the purpose of the visit is not a smoking-related problem. Such advice is not well received by patients, although according to standing procedures, it should be given to smokers at every visit [29-31]. Our findings also seem to suggest that it is difficult to establish one universal standard procedure for lifestyle intervention. What is more, actions in this direction must cater for the patients’ needs and take account of the way the healthcare system works. The very expectation that every time the smoker visits his GP the problem of smoking will be discussed is unrealistic due to lack of time. In a similar vein, bringing up the issue of smoking cessation persistently during every consultation, especially when it’s not connected with the purpose of the visit, may not only irritate the patient, but can also impede doctor-patient relationship. Nonetheless, it does not mean GPs can ignore anti-smoking intervention altogether.

Furthermore, infrequent pursuit of help from a GP has been reported by Hung et al., who conducted research among recent quitters. It has been established that recent quitters did not benefit from GPs’ advice as often as from other methods, such as cold turkey, gradual reduction before quitting and nicotine replacement therapy [45]. Also, a GP’s assistance has been perceived as less helpful than the above-mentioned methods. One reason for this is that patients rarely talked with their GP about smoking cessation as they did not always realize that the GP could help them*.* In addition, GPs did not intervene at all or only routinely advised smoking patients to quit. Likewise, in a study by Lambe and Collins, [41] GPs focused mainly on fear appeals, without offering any adequate support.

Patients’ ignorance about help in smoking cessation that GPs can offer could also result from the fact that the issue of quitting is rarely brought up by the GP during the visit. As some previous studies show, the major reasons behind poor lifestyle interventions are insufficient time and no reimbursement for undertaking such activities [41]. A similar situation can be found in Poland, where GPs admit many patients and are paid for treatment, not preventive, activities. It seems therefore that the role of the GP in smoking cessation has not been appreciated enough by patients. This is a missed opportunity, especially if we take into account a study by Murray et al., which shows that it is GP advice that acts as the most important trigger for unplanned quit attempts [46].

A limitation of this study is the relatively small number of interviews. Although we felt we had achieved saturation, there might be selection biases operating, such as an overrepresentation of patients with health problems due to the recruitment process conducted by the GPs. On the other hand, the recruitment of participants through the Institute of Sociology could have created a selection biases in terms of an overrepresentation of better-educated participants. Nevertheless, we still consider our sample to be complex enough to identify patients’ expectations and experiences of how their GPs deal with smoking cessation.

Another possible limitation are drawbacks resulting from focus group discussions, if compared with the presentation of experiences in individual interviews. These may embrace, for example, the striving for conformity in a group, which may prevent the participants from articulating their real personal experiences. A certain limitation in a group of patients who didn’t manage to stop smoking may be an increased need to make their GP a scapegoat for their failure to quit. Interaction among focus group participants may reaffirm this conviction because the GP could always try to do more for the patient to convince him to quit. This limitation is overcome to a certain extent by research among those who succeeded in smoking cessation and do not feel the need to look for the scapegoat.

Finally, the problem of the division of responsibility between the patient and the GP has not been addressed since this topic was not very much discussed in the groups.

### Implications for practice and further research

An important implication from this study is the necessity to bring the problem of smoking cessation to the GP, and encourage its discussion during patients’ visits. This requires, in turn, GP training in smoking cessation intervention. Such training programs should result in a situation where GPs act as facilitators and initiate a discussion about smoking cessation, provide a tailored approach to the patient, and use recent findings about the health hazards of smoking and potential methods of treatment.

Another important issue is the management of GPs’ work so that they can find time for smoking cessation interventions. Even a GP who is well-prepared and convinced of the righteousness of these interventions will not undertake them if there’s no time. Changes in work organization should limit GPs’ workload when it comes to treatment activities, and there’s also a need for a shift toward lifestyle interventions. In the case of Polish GPs this would relate to a cut in the number of patients GPs look after.

It is worth conducting future research to answer questions of what else may be the cause of GPs’ lack of interventions in smoking cessation, how to increase their number, and how this is going to influence patients’ attempts to quit smoking.

## Conclusion

While exploring the GP’s role in smoking cessation, the study revealed a big discrepancy between patients’ expectations and experiences. The smokers’ experiences of GPs’ aid in smoking cessation did not match their expectations because the topic of smoking was rarely discussed during visits and the doctor’s approach to the patient was perceived by the patients as routine. The participants of the study also observed that GPs had little time for any interventions in smoking cessation in their daily work and that the doctor-patient cooperation in the process of giving up smoking is poor.

Patients expect their GP to actively participate in smoking cessation. They also suggested that help should be adjusted to meet the smoker’s individual needs. For the majority of the investigated smokers this would mean GPs taking the lead in initiating conversations about smoking cessation, providing the patient with motivating information on quitting smoking, and assisting in therapy implementation and management.

Although the patients would like their GP to be more active in smoking cessation, to adopt a tailored approach, to provide them with evidence on the dangers of smoking, and cater to their individual needs, the results revealed that these desires are not often met. In summary, the GP should play a bigger role in smoking cessation.

## Competing interests

The authors declare that they have no competing interests.

## Authors’ contributions

KB and LM are responsible for the design of the study with comments from EP and SC. EP conducted the focus group interviews and transcribed them. KB, LM and AS categorized the data and discussed it with EP and SC. KB, AS and SC wrote the first draft of the manuscript and LM, EP commented on draft versions of the manuscript. All authors read and approved the final manuscript.

## Pre-publication history

The pre-publication history for this paper can be accessed here:

http://www.biomedcentral.com/1471-2296/14/159/prepub

## Supplementary Material

Additional file 1 Topic guide for focus group discussions. Click here for file
