# Supplementary material for: “What kind of general practitioner do I need for smoking cessation?” Results from a qualitative study in Poland
Source: BMC Fam Pract. 2013 Oct 20;14:159. doi: 10.1186/1471-2296-14-159 (PMC3853716; doi:10.1186/1471-2296-14-159)
Supplement: Additional file 1 — Topic guide for focus group discussions. [file 1471-2296-14-159-S1.doc]

Appendix 1. Topic guide for focus group discussions

1. Have you tried to give up smoking cigarettes before? How many times have you tried/did you try (before you succeeded)?
2. Did you/do you plan to prepare to stop smoking, or did/will it happen spontaneously?
3. If in the past, after quitting, you returned to smoking, what was the reason for this?
4. Have you ever talked with your GP about smoking cessation? If so, how did the talk go? What did the doctor say?
5. What do you think: should the GP help his/her patients quit smoking? Is it his/her responsibility?
6. What should the GP do to help the patient quit smoking?
7. What exactly do you want your GP to do or how do you want him/her to behave (in this respect)?
